# Metagenomics and metatranscriptomics reveal broadly distributed, active, novel methanotrophs in the Gulf of Mexico hypoxic zone and in the marine water column

**DOI:** 10.1093/femsec/fiac153

**Published:** 2022-12-15

**Authors:** Kathryn L Howe, Kiley W Seitz, Lauren G Campbell, Brett J Baker, J Cameron Thrash, Nancy N Rabalais, Mary-Kate Rogener, Samantha B Joye, Olivia U Mason

**Affiliations:** Department of Earth, Ocean, and Atmospheric Science, Florida State University, 32306, Tallahassee, United States; Department of Marine Science, Marine Science Institute, University of Texas at Austin, 78373, Port Aransas, United States; Department of Earth, Ocean, and Atmospheric Science, Florida State University, 32306, Tallahassee, United States; Department of Marine Science, Marine Science Institute, University of Texas at Austin, 78373, Port Aransas, United States; Department of Integrative Biology, University of Texas at Austin, 78712, Austin, United States; Department of Biological Sciences, University of Southern California, 90089, Los Angeles, United States; Department of Oceanography and Coastal Sciences, Louisiana State University, 70803, Baton Rouge, United States; Louisiana Universities Marine Consortium, 70344, Chauvin, United States; Department of Marine Sciences, University of Georgia, 30602, Athens, United States; Department of Marine Sciences, University of Georgia, 30602, Athens, United States; Department of Earth, Ocean, and Atmospheric Science, Florida State University, 32306, Tallahassee, United States

**Keywords:** hypoxic zone, metagenome assembled genome (MAG), metagenomics, metatranscriptomics, methane, methanotrophs

## Abstract

The northern Gulf of Mexico (nGOM) hypoxic zone is a shallow water environment where methane, a potent greenhouse gas, fluxes from sediments to bottom water and remains trapped due to summertime stratification. When the water column is destratified, an active planktonic methanotrophic community could mitigate the efflux of methane, which accumulates to high concentrations, to the atmosphere. To investigate the possibility of such a biofilter in the nGOM hypoxic zone we performed metagenome assembly, and metagenomic and metatranscriptomic read mapping. Methane monooxygenase (*pmoA*) was an abundant transcript, yet few canonical methanotrophs have been reported in this environment, suggesting a role for non-canonical methanotrophs. To determine the identity of these methanotrophs, we reconstructed six novel metagenome-assembled genomes (MAGs) in the Planctomycetota, Verrucomicrobiota and one putative Latescibacterota, each with at least one *pmoA* gene copy. Based on ribosomal protein phylogeny, closely related microbes (mostly from Tara Oceans) and isolate genomes were selected and co-analyzed with the nGOM MAGs. Gene annotation and read mapping suggested that there is a large, diverse and unrecognized community of active aerobic methanotrophs in the nGOM hypoxic zone and in the global ocean that could mitigate methane flux to the atmosphere.

## Teaser

Multi-omics approaches and analyses were used to identify and characterize novel, uncultured, non-canonical marine methanotrophs, including the pathways by which they carry out methane oxidation and their activity.

## Introduction

Methane, a potent greenhouse gas, at 150% of pre-industrial levels and rising (Saunois et al. 2016), has reached the highest level in the last 800 000 years (IPCC 2013). Emissions from marine environments are an important source of atmospheric methane, with the coastal and open ocean accounting for 1%–13% of natural emissions (Saunois et al. 2016). Methane produced by biological processes in sediments or in the water column can escape to the atmosphere (Reeburgh 2007, IPCC 2013, Kirschke et al. 2013, Saunois et al. 2020, Rosentreter et al. 2021). Conversely, microbial oxidation (aerobic and anaerobic) can capture methane and reduce atmospheric efflux. Methanotrophs account for 5% of the global methane sink (Saunois et al. 2020). While this takes place primarily in soils and marine sediments, methane oxidation also occurs in the marine water column (Reeburgh 2007, IPCC 2013, Kirschke et al. 2013, Saunois et al. 2020, Rosentreter et al. 2021).

In the Gulf of Mexico (GOM), methane enters the water column primarily from natural seeps, but also from drilling operations and accidents, such as the Deepwater Horizon oil spill in 2010, and from the flux of methane from anoxic sediments. When methane fluxes from sediments to the stratified water column in the northern GOM (nGOM) hypoxic zone, also called the “dead zone”, it remains trapped in bottom water due to density stratification during the summer (Rogener et al. 2021). Importantly, methane concentrations are high relative to concentrations expected from equilibrium with atmospheric concentrations in this coastal dead zone (Rogener et al. 2018), with concentrations ranging from 5 to 641 nM (Rogener et al. 2021). Overturning of the shallow, hypoxic bottom water in the nGOM dead zone can result in the flux of trapped gases (e.g. methane, nitrous oxide) to the atmosphere (Walker et al. 2010, Rogener et al. 2021). Thus, understanding the fate of this bottom water methane is important (Knief 2015) and requires determining the function of methanotrophs that could act as a biofilter in the water column.

Methanotrophs use methane as a carbon and energy source and are a subgroup of methylotrophs, microbes that degrade single-carbon compounds (Anthony 1982). Methanotrophs thrive at oxic-anoxic interfaces (Hanson and Hanson 1996, Knief 2015), but exist across a range of environments, reflecting their physiological diversity. For example, Kalyuzhnaya et al. (2019) suggested methanotrophic bacteria are ubiquitous in the environment as both planktonic microbes and as symbionts of several organisms, including mussels, snails, sponges and tubeworms (reviewed in Dubilier et al. 2008).

Canonical aerobic methanotrophs, described as early as 1906 (Söhngen 1906), are categorized based on morphological characteristics, such as membrane type, physiology, methane oxidation pathway, methane monooxygenase sequence similarity and 16S rRNA phylogeny (Hanson and Hanson 1996, Kalyuzhnaya et al. 2019). In the marine environment, canonical aerobic marine methanotrophs are mainly affiliated with Proteobacteria, and more specifically the Gammaproteobacteria and Alphaproteobacteria (Hanson and Hanson 1996). More recently, methylotrophy is viewed as a modular genetic system encompassing an indeterminate number of gene combinations that enable transformation of single-carbon compounds into biomass and/or other metabolically useful compounds (Chistoserdova 2011).

The first step in aerobic methane oxidation (MOx) is the conversion of methane to methanol via methane monooxygenase (particulate or soluble, pMMO/sMMO) (Hanson and Hanson 1996). Virtually all methanotrophs code for pMMO (Murrell et al. 2000); however, only a few methanotrophs encode sMMO, which is utilized in copper-deficient environments (Semrau et al. 2010). The ubiquity of pMMO in the marine environment makes the alpha subunit of particulate methane monooxygenase (*pmoA*) a robust functional marker gene to identify methanotrophs (Mcdonald and Murrell 1997). Crespo-Medina et al. (2014) used this approach to identify a diversity of methanotrophs that responded to the input of methane during the Deepwater Horizon oil spill in the Gulf of Mexico.

**Figure 1.**
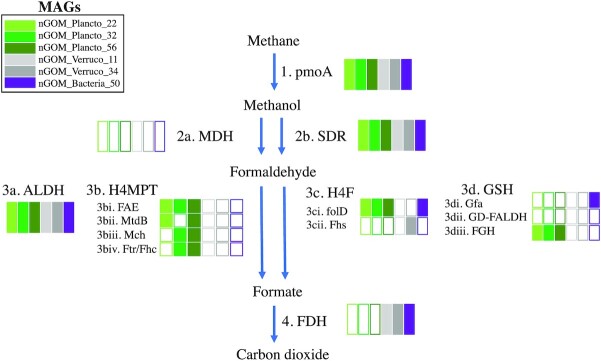
Schematic of the aerobic methane oxidation pathway. The colored boxes, which are color coded for taxonomy, correspond to the presence (colored boxes) or absence (empty boxes) of genes coding for enzymes necessary for these steps in the nGOM MAGs. Blue arrows indicate oxidation steps. The abbreviations shown in the figure are as follows: pmoA/AMO: particulate methane monooxygenase/ammonia monooxygenase; MDH: methanol dehydrogenase; SDR: short-chain alcohol dehydrogenase; ALDH: aldehyde dehydrogenase; H4MPT: tetrahydromethanopterin pathway; FAE: formaldehyde-activating enzyme; MtdB: methylene tetrahydromethanopterin dehydrogenase; Mch: methenyl-tetrahydromethanopterin cyclohydrolase; Ftr/Fhc: formylmethanofuran-tetrahydromethanopterin formyltransferase; H4F: tetrahydrofolate pathway; folD: tetrahydrofolate dehydrogenase/cyclohydrolase; Fhs: formate–tetrahydrofolate ligase; GSH: glutathione-dependent pathway; Gfa: glutathione-dependent formaldehyde activating enzyme; GD-FALDH: glutathione-dependent formaldehyde dehydrogenase; FGH: Formyl-glutathione hydrolase; FDH: formate dehydrogenase.

Fewer studies have looked beyond *pmoA* and evaluated the complete four-step pathway that is utilized by aerobic, planktonic methanotrophs. Methane oxidation proceeds via its enzymatic conversion to methanol (discussed above) followed by transformation of methanol to formaldehyde, which is mediated by methanol dehydrogenase (MDH) or a short-chain alcohol dehydrogenase (SDR). Formaldehyde is a central intermediate for methanotrophs, assimilated or oxidized to formate through multiple pathways, and formate is then assimilated via the serine pathway or is oxidized to carbon dioxide (CO_2_) via formate dehydrogenase (FDH) (Hanson and Hanson 1996, Vorholt 2002, Chistoserdova et al. 2009) (Fig. 1). In this study, we assessed the complete pathways for methane oxidation in multiple novel genomes. We would also note that some methanotrophs, such as *Candidatus Methylomirabilis oxyfera*, and Proteobacteria and Verrucomicrobiaota, can assimilate CO_2_, utilizing the serine or Calvin-Benson-Bassham cycles (Khadem et al. 2011, Rasigraf et al. 2014), which were also evaluated in our study.

Despite the high methane concentrations in the nGOM dead zone, microbial ecology studies using iTag sequencing of 16S rRNA gene amplicons have reported low to undetectable abundances of canonical methanotrophs in this environment (Gillies et al. 2015, Campbell et al. 2019). Observations of high methane concentrations and oxidation rates in the nGOM dead zone (Kelley 2003, Rogener et al. 2018, 2021), coupled with the lack of canonical methanotrophs, suggested a role for an active planktonic methanotrophic community. Here, we sought to determine what planktonic microorganisms could carry out methane oxidation (MOx) through assembly of metagenomes and metabolic reconstruction. Further, to determine which microbes were actively mediating MOx, metatranscriptomic reads were mapped to our metagenome-assembled genomes (MAGs). Finally, complete to near complete genomes of close relatives of those nGOM microbes were identified and included in this analysis to evaluate active MOx in the 2013 nGOM dead zone and more broadly in the marine environment. Inclusion of these close relatives was done for two reasons. First, the nGOM MAGs assembled and presented herein were not complete, which presents challenges when trying to determine the full genetic repertoire encoded in these uncultured microbes. For example, co-analysis of relatives with complete to near-complete genomes provided information on genes that may have been missing due to an incomplete genome assembly in the nGOM MAGs. Second, the additional microbes were sampled from the global ocean, which expands our understanding of microbes mediating MOx outside of the nGOM.

## Materials and Methods

### Sample location and collection

Samples were collected at the oxygen minimum zone (OMZ) on the R/V *Pelican* in the dead zone along transects running perpendicular to the hypoxic zone starting from the mouth of the Mississippi River and ending near the Louisiana-Texas border in 2013 (Gillies et al. 2015) (see Supp. Fig. 1). The depth of water sample collection ranged from 6 to 35 meters below sea level, with an average sampling depth of 16 m, with up to 10 L collected at each location (Gillies et al. 2015). Oxygen concentrations were determined with a CTD oxygen sensor and calibrated using the Winkler method (Gillies et al. 2015). For our study, the samples O_D1, H_D2, H_D3, O_E2 and H_E4 (O means oxic, H means hypoxic) were selected for “omics” sequencing (Supp. Fig. 1).

### Microbial sampling and DNA and RNA extraction

Samples for microbial analysis were collected by filtering up to 10 L of seawater through 2.7-μm pre-filters and then onto 0.22-μm Sterivex filters, which were preserved in RNAlater and immediately frozen. Details regarding DNA and RNA extraction, sequencing and analysis can be found in Gillies et al. (2015) and Thrash et al. (2017). Briefly, DNA and RNA were extracted directly off of the frozen, RNAlater-preserved filters by placing half of a Sterivex filter in a Lysing matrix E glass/zirconia/silica beads tube (MP Biomedicals, Santa Ana, CA, USA) following the protocol in Gillies et al. (2015) that combines phenol: chloroform: isoamyalcohol (25 : 24 : 1) and bead beating. Genomic DNA and RNA were purified using a QIAGEN (Valencia, CA, USA) AllPrep DNA/RNA Kit. DNA was quantified using a Qubit2.0 Fluorometer (Life Technologies, Grand Island, NY, USA). RNA quality was analyzed using an Agilent TapeStation with an RNA integrity number (RIN) (16S/23S rRNA gene ratio) to assess degradation (scale of 1 to 10, 10 being undegraded RNA). RNA with RIN scores of ≥8 was chosen for metatranscriptomic sequencing. Prior to sequencing, rRNA was subtracted from total RNA using a Ribo-Zero kit (Illumina) and mRNA was reverse transcribed to cDNA, as described in Mason et al. (2012).

### 16S rRNA gene sequence data

16S rRNA gene data from the 2013 and 2014 nGOM dead zone presented in Gillies et al. (2015) and Campbell et al. (2019) were used to determine the abundances of canonical methanotrophs (Tavormina et al. 2008). In these datasets, the relative abundances of Methylococcales, specifically *Methylobacter, Methylococcus* and *Methylomicrobium* in the Gammaproteobacteria, and *Methylosinus* and *Methylocystis* in the Alphaproteobacteria, were determined.

### Metagenome and metatranscriptome sequencing and analyses

Metagenomes and metatranscriptomes were sequenced separately using six samples per lane with the Illumina HiSeq 2000, to produce 100 bp, paired-end reads (Thrash et al. 2017). Co-assembly of the metagenomes is described in Thrash et al. (2017). Briefly, reads were quality-filtered, pooled and assembled using IDBA-UD (Peng et al. 2012), binning was performed using emergent self-organizing maps (ESOM) of contigs ≥5 kb, bin quality control was done with CheckM (Parks et al. 2015) and annotation was carried out with the Integrated Microbial Genomes (IMG) (Markowitz et al. 2014), which resulted in 70 MAGs published previously (Thrash et al. 2017, 2018), plus an additional seven MAGs that were assembled but not published. An *in silico* search for *pmoA* annotations in all MAGs was carried out using the IMG annotation pipeline v. 5.0.0 (https://img.jgi.doe.gov/docs/pipelineV5/) and searched for functions and genes within these annotations, with six nGOM MAGs identified as having this gene. Of these six MAGs, Thrash et al. (2017) presented an analysis of only the unclassified Bacteria, putatively identified as Latescibacterota (bin 50) analyzed here, and while its *pmoA* gene was annotated and reported, this finding was not discussed. Taxonomic classification of the nGOM MAGs was assigned using GTDB-Tk (v. 1.0.2) (Parks et al. 2018, Chaumeil et al. 2019).

Five unassembled metatranscriptomes and five unassembled metagenomes from Thrash et al. (2017) were mapped to the six nGOM MAGs that had at least one *pmoA* copy using Bowtie2 (Langmead and Salzberg 2012). Prior to mapping, ribosomal RNA was subtracted from the metatranscriptome reads using riboPicker v. 0.4.3 with the default settings (Schmieder et al. 2012). On average, 8% of the metatranscriptomic reads were ribosomal RNA that were subsequently removed *in silico* with riboPicker.

**Figure 2.**
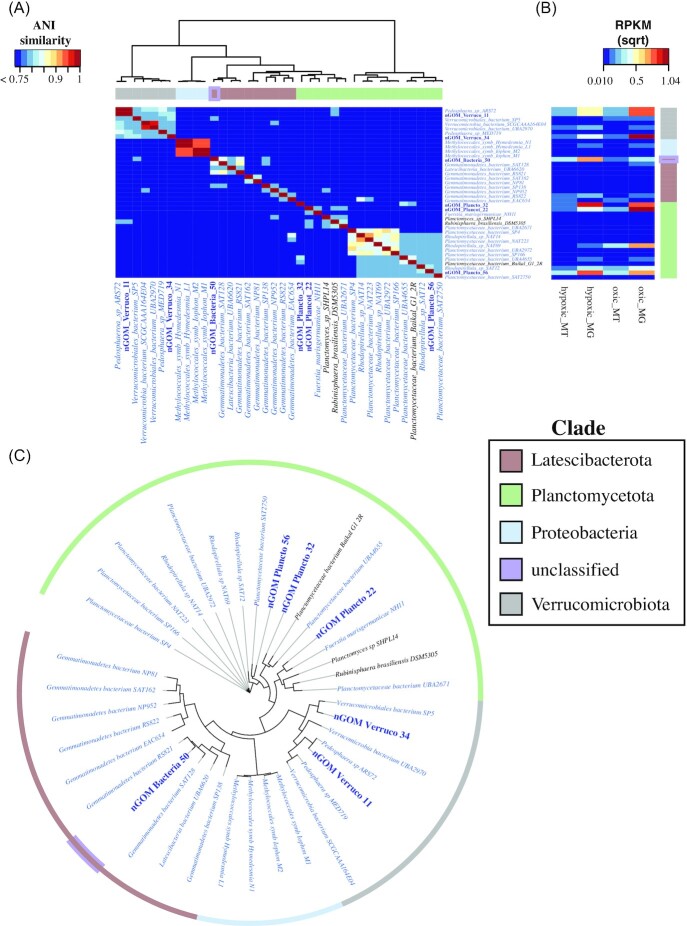
ANI heatmap **(A)**, square root RPKM abundance **(B)** and phylogenetic tree **(C)** of the 38 genomes. Genome names are color-coded blue and bold (nGOM MAGs), black (non-marine) and blue (marine). Only ANI above 75% is shown in 2A. The clade color code for nGOM Bacteria 50 is brown with a purple border to indicate that is putatively identified as Latescibacteria, but is herein classified as unknown.

Using the GTDB-tk ribosomal protein phylogenetic tree, close relatives of the nGOM MAGs were identified using phylogeny with monophyly and branch length as decision-making criteria for inclusion (see Supp. Fig. 2). All of these close relatives encoded *pmoA*. Four additional Proteobacteria MAGs were included here because: (1) they represent a canonical methanotroph clade; (2) they encode *pmoA*, as the nGOM MAGs do; and (3) they were also sampled from the GOM (deepwater asphalt seeps) (Rubin-Blum et al. 2019). All together, we assessed six nGOM MAGs, 28 non-nGOM MAGs and four canonical southern GOM MAGs that were included in all subsequent analyses (Table 1). A second phylogenetic tree (Fig. 2C) was constructed with the 38 genomes analyzed herein using the FastTree (v. 2) (Price et al. 2010) implementation in Anvi'o (v. 6.2) (Eren et al. 2015) with all 71 single-copy core genes (ribosomal and non-ribosomal) in the default bacterial collection and visualized using iTOL (v. 6) (Letunic and Bork 2021).

**Table 1.** Microbial taxonomy, study and sample information for MAGs and genomes from cultivated representatives. The nGOM MAGs are shown in bold text.

Genome	Taxonomy	Reference	Sample location	GenBank accession*
Methylococcales symb Hymedesmia L1	Proteobacteria;Gammaproteobacteria;Methylococcales;Methylomonadaceae	Rubin-Blum et al. 2019	GOM (southern)	GCA_003666335.1
Methylococcales symb Hymedesmia N1	Proteobacteria;Gammaproteobacteria;Methylococcales;Methylomonadaceae	Rubin-Blum et al. 2019	GOM (southern)	GCA_003666385.1
Methylococcales symb Iophon M1	Proteobacteria;Gammaproteobacteria;Methylococcales;Methylomonadaceae	Rubin-Blum et al. 2019	GOM (southern)	GCA_003666325.1
Methylococcales symb Iophon M2	Proteobacteria;Gammaproteobacteria;Methylococcales;Methylomonadaceae	Rubin-Blum et al. 2019	GOM (southern)	GCA_003666345.1
**nGOM Plancto 22**	Planctomycetota;Planctomycetes;Planctomycetales;Planctomycetaceae	this study	GOM (northern)	2693429805 (IMG ID)
**nGOM Plancto 32**	Planctomycetota;Planctomycetes;Planctomycetales;Planctomycetaceae	this study	GOM (northern)	2651870066 (IMG ID)
**nGOM Plancto 56**	Planctomycetota;Planctomycetes;Planctomycetales;Planctomycetaceae	this study	GOM (northern)	2651870065 (IMG ID)
Fuerstia marisgermanicae NH11***	Planctomycetota;Planctomycetes;Planctomycetales;Planctomycetaceae	Kohn et al. 2016	Wadden Sea Germany; crab shell	GCA_001983935.1
Planctomyces sp SHPL14***	Planctomycetota;Planctomycetes;Planctomycetales;Planctomycetaceae	NA^	sugar beet rhizosphere	GCA_001610835.1
Planctomycetaceae bacterium Baikal G1 2R	Planctomycetota;Planctomycetes;Pirellulales;UBA1268	Cabello-Yeves et al. 2018	Lake Baikal (Siberia, Russia)	GCA_002737405.1
Planctomycetaceae bacterium NAT223	Planctomycetota;Planctomycetes;Pirellulales;Pirellulaceae	Tully et al. 2018	**Eastern Tropical North Pacific (TARA_137)	GCA_002705165.1
Planctomycetaceae bacterium SAT2750	Planctomycetota;Planctomycetes;Pirellulales;Pirellulaceae	Tully et al. 2018	**Equatorial Tropical Pacific (TARA_128)	GCA_002708345.1
Planctomycetaceae bacterium SP166	Planctomycetota;Planctomycetes;Pirellulales;Pirellulaceae	Tully et al. 2018	**Equatorial Tropical Pacific (TARA_128)	GCA_002709045.1
Planctomycetaceae bacterium SP4	Planctomycetota;Planctomycetes;Pirellulales;Pirellulaceae	Tully et al. 2018	**coast of Chile (TARA_093)	GCA_002717145.1
Planctomycetaceae bacterium UBA2671	Planctomycetota;Planctomycetes;Planctomycetales;Planctomycetaceae	Parks et al. 2017	NA (marine)	GCA_002359185.1
Planctomycetaceae bacterium UBA2972	Planctomycetota;Planctomycetes;Pirellulales;Pirellulaceae	Parks et al. 2017	NA (marine)	GCA_002348315.1
Planctomycetaceae bacterium UBA4655	Planctomycetota;Planctomycetes;Pirellulales;UBA1268	Parks et al. 2017	NA (freshwater)	GCA_002405515.1
Rhodopirellula sp NAT14	Planctomycetota;Planctomycetes;Pirellulales;Pirellulaceae	Tully et al. 2018	**Arabian Sea (TARA_036)	GCA_002698965.1
Rhodopirellula sp NAT69	Planctomycetota;Planctomycetes;Pirellulales;Pirellulaceae	Tully et al. 2018	**Arabian Sea (TARA_036)	GCA_002701385.1
Rhodopirellula sp SAT12	Planctomycetota;Planctomycetes;Pirellulales;Pirellulaceae	Tully et al. 2018	**Arabian Sea (TARA_036)	GCA_002714565.1
Rubinisphaera brasiliensis DSM5305	Planctomycetota;Planctomycetes;Planctomycetales;Planctomycetaceae	Scheuner, C. et al. 2014	Water from salt pit, Lagoa Vermelha, Brazil	GCA_000165715.2
Gemmatimonadetes bacterium EAC654	Latescibacterota;UBA2968;UBA8231;UBA8231	Tully et al. 2018	**Eastern Tropical North Pacific (TARA_137)	GCA_002693325.1
Gemmatimonadetes bacterium NP81	Latescibacterota;UBA2968;UBA8231;UBA8231	Tully et al. 2018	southern coast of Africa (TARA_065)	GCA_002726335.1
Gemmatimonadetes bacterium NP952	Latescibacterota;UBA2968;UBA8231;UBA8231	Tully et al. 2018	Red Sea (TARA_032)	GCA_002725915.1
Gemmatimonadetes bacterium RS821	Latescibacterota;UBA2968;UBA8231;UBA8231	Tully et al. 2018	southern coast of Africa (TARA_065)	GCA_002727195.1
Gemmatimonadetes bacterium RS822	Latescibacterota;UBA2968;UBA8231;UBA8231	Tully et al. 2018	southern coast of Africa (TARA_065)	GCA_002724215.1
Gemmatimonadetes bacterium SAT128	Latescibacterota;UBA2968;UBA2968;GCA-2 709 665	Tully et al. 2018	**Equatorial Tropical Pacific (TARA_128)	GCA_002714465.1
Gemmatimonadetes bacterium SAT162	Latescibacterota;UBA2968;UBA2968;UBA2968	Tully et al. 2018	**Equatorial Tropical Pacific (TARA_128)	GCA_002712305.1
Gemmatimonadetes bacterium SP138	Latescibacterota;UBA2968;UBA2968;UBA2968	Tully et al. 2018	**Equatorial Tropical Pacific (TARA_128)	GCA_002709665.1
Latescibacteria bacterium UBA6620	Latescibacterota;UBA2968;UBA2968;UBA2968	Parks et al. 2017	NA (marine)	GCA_002433075.1
**nGOM Bacteria 50**	unclassified;unclassified;unclassified;unclassified	Thrash et al. 2017	GOM (northern)	2693429804 (IMG ID)
**nGOM Verruco 11**	Verrucomicrobiota;Verrucomicrobiae;Verrucomicrobiales;unclassified	this study	GOM (northern)	2651870083 (IMG ID)
**nGOM Verruco 34**	Verrucomicrobiota;Verrucomicrobiae;Verrucomicrobiales;unclassified	this study	GOM (northern)	2651870086 (IMG ID)
Pedosphaera sp ARS72	Verrucomicrobiota;Verrucomicrobiae;Pedosphaerales;AAA164-E04	Tully et al. 2018	**Arabian Sea (TARA_036)	GCA_002686885.1
Pedosphaera sp MED719	Verrucomicrobiota;Verrucomicrobiae;Pedosphaerales;AAA164-E04	Tully et al. 2018	Mediterranean Sea (TARA_018)	GCA_002690655.1
Verrucomicrobia bacterium SCGCAAA164E04	Verrucomicrobiota;Verrucomicrobiae;Pedosphaerales;AAA164-E04	Stepanauskas et al. 2013	Gulf of Maine	GCA_000383715.1
Verrucomicrobia bacterium UBA2970	Verrucomicrobiota;Verrucomicrobiae;Pedosphaerales;AAA164-E04	Parks et al. 2017	NA (marine)	GCA_002348345.1
Verrucomicrobiales bacterium SP5	Verrucomicrobiota;Verrucomicrobiae;Pedosphaerales;AAA164-E04	Tully et al. 2018	**Arabian Sea (TARA_036)	GCA_002715965.1

*Unless otherwise indicated

** site where annual mean oxygen <2 ml/l (Pesant et al. 2015)

*** Cultured microbe

^submitter is Victor de Jager (v.dejager@nioo.knaw.nl)

Average nucleotide identity (ANI) was determined using Sourmash with -containment -ani -ksize 31 (Brown and Irber 2016). All genomes were analyzed using Anvi’o (v. 6.2) (Eren et al. 2015). Genes were annotated with functions by using the “anvi-run-pfams” and “anvi-run-ncbi-cogs” with the Pfam (Bateman et al. 2004) and COGs (Tatusov et al. 2000) databases. Outside of Anvi'o, the Pfam and COGs annotations were verified by reviewing annotations using blastx with DIAMOND (v. 0.9.30) (Buchfink et al. 2014) and NCBI's non-redundant RefSeq protein dataset (accessed from NCBI on 03/10/2020) (Tatusova et al. 2016). Functional annotation using COGs and Pfams databases, or blastx with the non-redundant RefSeq protein database, were confirmed if two of three database annotations agreed. These annotations were then used to identify the gene and transcript sequences comprising the different modules of the methane oxidation pathways. Using Bowtie2, metatranscriptome and metagenome reads were mapped to the annotated genes in each genome to determine abundance and expression by calculating reads per kilobase per million (RPKM) values following Thrash et al. (2017). RPKM values were also calculated to determine genome abundances and activity by mapping metagenome and metatranscriptome reads to each genome. To classify functions that were part of the full pangenome core, “anvi-get-enriched-functions-per-pan-group” was used in Anvi'o to identify individual clade cores using the ribosomal protein tree phylogeny (see above). This command was also used to determine what functions were statistically enriched in each clade by using a generalized linear model with logit linkage function.

## Results

### Chemistry

Of the five samples from the nGOM hypoxic zone selected for metagenomic and metatranscriptomic sequencing (Supp. Fig. 1) that were originally reported on in Thrash et al. (2017), oxygen concentrations were reported in Gillies et al. (2015) and additionally in Thrash et al. (2017) for the five metagenomic and metatranscriptomic samples. Of the five samples selected, two were oxic, O_D1 and O_E2 (4.12 and 2.64 mg L^-1^ dissolved oxygen (O_2_), respectively), and three were hypoxic, H_D3, H_D2 and H_E4 (0.4, 0.33 and 0.31 mg L^-1^ O_2_, respectively). Sampling at the same time and at directly adjacent sites, Rogener et al. (2021) found that methane concentrations were negatively correlated with O_2_ concentrations and reported MOx rates as high as 192 nmol L^−1^d^−1^ and depth-integrated methane oxidation rates (Fmox) of 0.2 µmol m^−2^d^−1^ in 2013, which was lower than Fmox in the other two years they analyzed (2.4 in 2015 and 322 in 2016). The estimated atmospheric flux of methane was also lowest in 2013 compared with the other two years (27 vs. 97 and 278 µmol m^−2^d^−1^) (Rogener et al. 2021).

### Paucity of canonical methanotrophs in a legacy 16S rRNA gene sequence dataset

Reanalysis of the 16S rRNA gene amplicon sequence data from the 2013 dead zone that was presented in Gillies et al. (2015) and the 2014 dead zone reported on in Campbell et al. (2019) revealed that the relative proportions of canonical methanotrophs (Tavormina et al. 2008) were very low. Specifically, Methylococcales (Gammaproteobacteria) comprised an average of 0.07% of the population in the oxic samples and an average of 0.09% in hypoxic samples. Other canonical methanotrophs, such as *Methylobacter, Methylococcus* and *Methylomicrobium* in the Gammaproteobacteria, and *Methylosinus* and *Methylocystis* in the Alphaproteobacteria, were either not observed or were below 0.004% relative abundance in samples collected at any site in either year.

### Taxonomy, phylogenetic relatedness, abundance and activity

**Figure 3.**
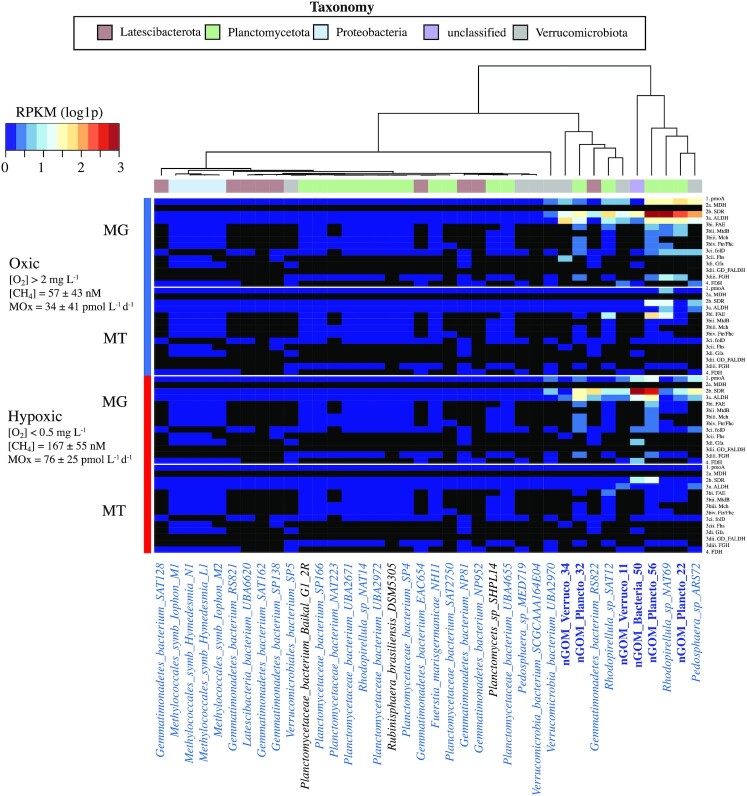
Heatmap of abundance (metagenome, MG) and expression (metatranscriptome, MT) of genes involved in aerobic marine methane oxidation. Genome names at the bottom are color-coded blue and bold (nGOM MAGs), black (non-marine) and blue (marine). Genes not encoded in a particular genome are shown as a black box in the figure. The FDH of nGOM Verruco 34 was annotated only in IMG and not confirmed by COGs and Pfam annotations and is therefore shown as not annotated in this figure.

Six nGOM MAGs were co-assembled from the 2013 dead zone metagenome data (Thrash et al. 2017) with average genome completeness of 58% and 2.2% contamination (Table 2). Five of these microbes were classified as Planctomycetota (three MAGs) and Verrucomicrobiota (two MAGs). The remaining MAG was putatively classified as Latescibacterota (formerly WS3) by ribosomal protein phylogeny, but as PAUCF/SAUL by 16S rRNA genes and amplicon data (Thrash et al. 2017), so is herein designated as unclassified. These nGOM MAGs all encoded genes for PmoA, part of the pMMO enzyme necessary for methane oxidation to methanol, as well as a full or partial MOx pathway (Fig. 1). In addition to these six nGOM MAGS, relatives chosen using the GTDB phylogenetic tree monophyly and branch length as criteria for inclusion, resulted in 28 non-nGOM genomes being co-analyzed, with an additional four genomes included that represented canonical methanotrophs. Of these 32 additional genomes that were co-analyzed here, 92% were sampled from the marine environment (Table 1, Fig. 2C) and all encoded PmoA and a partial to full MOx pathway (Fig. 3).

**Table 2.** Statistics for MAGs and genomes from cultivated representatives. The nGOM MAGs are shown in bold text.

Genome	Completeness	Contamination	Contig count	Scaffold count	Genome size	% GC
Methylococcales symb Hymedesmia L1	94.96%	0.29%	86	84	2 010 086	37.83
Methylococcales symb Hymedesmia N1	96.45%	0.31%	64	63	2 219 015	37.76
Methylococcales symb Iophon M1	95.59%	0.47%	123	123	2 092 041	37.73
Methylococcales symb Iophon M2	94.55%	0.46%	128	126	2 006 182	37.67
**nGOM Plancto 22**	46.41%	2.22%	3330	688	3 841 030	55.52
**nGOM Plancto 32**	52.07%	7.02%	2123	362	2 492 421	62.50
**nGOM Plancto 56**	83.84%	2.56%	3836	322	4 807 217	51.78
Fuerstia marisgermanicae NH11**	96.60%	1.11%	1	1	8 920 478	55.90
Planctomyces sp SHPL14**	98.89%	2.22%	1	1	8 442 773	65.56
Planctomycetaceae bacterium Baikal G1 2R	93.04%	13.22%	98	98	3 383 196	57.49
Planctomycetaceae bacterium NAT223	67.86%	2.60%	157	157	2 943 028	48.09
Planctomycetaceae bacterium SAT2750	56.03%	0.00%	102	102	1 910 127	58.19
Planctomycetaceae bacterium SP166	92.87%	0.00%	69	69	4 535 358	46.53
Planctomycetaceae bacterium SP4	70.54%	0.00%	115	115	4 424 693	47.85
Planctomycetaceae bacterium UBA2671	95.56%	0.00%	330	127	6 652 450	48.68
Planctomycetaceae bacterium UBA2972	95.87%	0.11%	401	253	4 362 043	49.07
Planctomycetaceae bacterium UBA4655	90.08%	0.00%	626	320	3 844 742	68.04
Rhodopirellula sp NAT14	89.32%	2.30%	192	192	5 424 119	46.52
Rhodopirellula sp NAT69	97.58%	0.00%	100	100	4 890 980	48.08
Rhodopirellula sp SAT12	94.64%	1.18%	119	119	4 537 602	48.43
Rubinisphaera brasiliensis DSM5305	95.56%	2.22%	1	1	6 006 602	56.45
Gemmatimonadetes bacterium EAC654	89.50%	7.14%	166	166	3 573 524	41.14
Gemmatimonadetes bacterium NP81	83.62%	4.92%	279	279	5 970 290	63.93
Gemmatimonadetes bacterium NP952	85.61%	5.01%	293	293	3 810 359	62.74
Gemmatimonadetes bacterium RS821	86.81%	1.10%	38	38	5 422 662	59.09
Gemmatimonadetes bacterium RS822	93.59%	3.30%	266	266	5 850 589	62.44
Gemmatimonadetes bacterium SAT128	97.74%	8.24%	78	78	5 214 681	56.54
Gemmatimonadetes bacterium SAT162	90.11%	2.75%	146	146	4 765 829	60.21
Gemmatimonadetes bacterium SP138	94.44%	4.40%	84	84	5 266 773	52.96
Latescibacteria bacterium UBA6620	95.60%	1.10%	151	61	5 642 168	59.49
**nGOM Bacteria 50**	84.80%	1.40%	5484	455	5 346 994	57.52
**nGOM Verruco 11**	51.72%	0%	1636	327	1 757 369	53.66
**nGOM Verruco 34**	27.77%	0%	1128	222	1 175 995	58.54
Pedosphaera sp ARS72	97.41%	7.37%	140	140	4 267 059	53.30
Pedosphaera sp MED719	86.49%	2.27%	35	35	4 083 318	47.78
Verrucomicrobia bacterium SCGCAAA164E04	71.90%	2.36%	222	218	3 949 105	47.70
Verrucomicrobia bacterium UBA2970	86.61%	2.70%	779	500	4 780 578	57.17
Verrucomicrobiales bacterium SP5	95.27%	2.36%	101	101	6 081 546	56.46

** Cultured microbe

The ribosomal protein tree phylogeny revealed the most similar microbes to nGOM MAGs were largely from globally distributed TARA Oceans samples (Tully et al. 2018), many of which were low oxygen marine environments (Table 1). For example, the nGOM Planctomycetota MAGs were most similar to Planctomycetales and Pirellulales obtained from sites with annual mean oxygen less than 2.66 mg L^−1^ (Pesant et al. 2015) (Table 1) (see Fig. 2C and Supp. Fig. 2, Table 1 for full taxonomy, Table 2 for genome statistics). Similarly, the nGOM Verrucomicrobiota MAGs were most similar to members of the Pedosphaerales (see Fig. 2C and Supp. Fig. 2, Table 1 for full taxonomy, Table 2 for genome statistics) sampled from TARA Oceans samples (Tully et al. 2018), two of which were collected from Arabian Sea sites with annual mean oxygen less than 2.66 mg L^−1^ (Pesant et al. 2015). The nGOM Bacteria 50 was most similar to Latescibacterota in the UBA2968 and UBA8231 orders (see Fig. 2C and Supp. Fig. 2, Table 1 for full taxonomy, Table 2 for genome statistics) primarily from TARA Oceans samples in the Eastern Tropical North Pacific, the coast of southern Africa and the Red Sea (Tully et al. 2018).

In addition to analyzing ribosomal protein tree phylogenetic relationships, ANI analysis was carried out, revealing that nGOM MAGs were more similar to non-nGOM microbes that were sampled from the global ocean than they were to one another (Fig. 2A and C). This is best exemplified by nGOM Verruco 11 and 34 with an ANI of 79%, while nGOM Verruco 11 had an ANI of 98% with *Pedosphaera sp ARS72*(Fig. 2A and C), suggesting that the latter pair could be the same species (Goris et al. 2007, Richter and Rosselló-Móra 2009, Kim et al. 2014). Similarly, the nGOM Planctomycetota MAGs were <75% similar to one another, but up to 80% similar to non-nGOM Planctomycetaceae, such as nGOM Verruco 34 and bacterium UBA4655 (Fig. 2A and C). The nGOM Bacteria 50 is the only representative of the unclassified, putative Latescibacterota in the nGOM data, thus its ANI was highest with non-GOM genomes, such as that of *Gemmatimonadetes bacterium RS821*(ANI = 82%) and *Gemmatimonadetes bacterium SAT128*(ANI = 81%; Fig. 2A).

To determine genome abundance and activity, DNA and RNA reads were recruited to each of the 38 genomes. On average, the nGOM MAGs recruited the greatest number of DNA and RNA reads of the 38 genomes (Fig. 2B), with DNA RPKM values ranging from an average of 0.1 to 1.07. For example, nGOM Plancto 22 and 56 had DNA RPKM values up to 1.50, while the nGOM Verruco 34 maximum was 2.09 and nGOM Bacteria 50 was up to 1.15 (Fig. 2B). There were some exceptions to this, with the non-nGOM *Rhodopirellula sp NAT69*having DNA RPKM values of up to 1.23. No other genomes had DNA RPKM values >1.0. As a point of comparison with Thrash et al. (2017), MAGs from the nGOM, our nGOM MAGs, averaged 0.46 RPKM, while those in Thrash et al. (2017) ranged in abundance from 13.9 RPKM for Euryarchaeoata to similar abundances to our MAGs, particularly the Candidate Phyla. The four canonical methanotroph Proteobacteria were classified as Methylococcales (see Table 1 for full taxonomy, Table 2 for genome statistics) and are common methane-oxidizing symbionts of various marine organisms (Rubin-Blum et al. 2019). These MAGs are included here to represent canonical methanotrophs, not because of close phylogenetic relationships with the nGOM MAGs (Fig. 2C). While these genomes recruited DNA reads from each sample, RPKM values were lower than the nGOM MAGs and other microbes discussed previously.

The RNA read recruitment pattern was generally the same as that for DNA, with the nGOM MAGs presented herein recruiting the greatest number of RNA reads of the microbes analyzed here, with RPKM values up to 0.34 (Fig. 2B). However, three non-GOM Planctomycetota and Verrucomicrobiota from TARA Oceans samples (Tully et al. 2018), collected from Arabian Sea sites with an annual mean oxygen of less than 2.66 mg L^−1^ (Pesant et al. 2015), had similar RNA RPKM values as the nGOM MAGS, with RPKM values up to 0.30. The nGOM Bacteria 50 recruited RNA reads from each sample, with a maximum RPKM of 0.19 (Fig. 2B). Thus, nGOM Bacteria 50 was active, and for sample H_E4, recruited the greatest number of RNA reads of any of the 38 microbes analyzed herein, which agrees with Thrash et al. (2017), who reported this as one of the most active microbes in the 2013 dead zone analysis based on its high relative DNA to RNA recruitment rank and cytochrome c oxidase expression. All four canonical methanotrophs recruited low levels of RNA reads, relative to the other 34 genomes, particularly the nGOM MAGs.

### The pangenome and clade core functions

The microbes analyzed herein are united in their functional potential to oxidize methane, which is a novel metabolism for many of these taxa, and none are recognized as marine methanotrophs. Therefore, we sought to determine their pangenome core functions. These methanotroph core functions were defined as having the same annotation in a minimum of two out of the three results obtained using COGs and Pfams databases, or blastx with the non-redundant RefSeq protein database from NCBI, as well as being found in all 38 genomes. We also defined clade-specific core functions the same way.

The full pangenome core contained 26 functions that included mostly housekeeping genes, ABC transporters and aromatic amino acid synthesis enzymes (Supp. Table 1). However, three core functions were worth noting: (1) NAD-dependent aldehyde dehydrogenase (ALDH), which is a non-specific enzyme in numerous pathways such as C1 and alkane degradation, including the formaldehyde oxidation step of the methane oxidation pathway (Patel et al. 1979, Anthony 1982); (2) NAD(P)-dependent dehydrogenase, SDR family protein, which can be utilized in a variety of transformations, including the second step of methane oxidation (methanol conversion to formaldehyde) (Arfman et al. 1997, Guo et al. 2019); and (3) *pmoA* (Supp. Table 1).

In the individual clades, 114 core functions were determined for the Planctomycetota, which included housekeeping genes, such as those for tRNA synthesis, outer membrane proteins and iron-containing alcohol dehydrogenases (Supp. Table 2). Two functions (tRNA-modifying enzymes and nucleotide sugar biosynthesis) were statistically enriched in the Planctomycetota (Supp. Table 2). In the verrucomicrobial clade, 89 functions were determined to be core (Supp. Table 3), most of which were housekeeping genes, including ribosomal proteins and phosphorylation enzymes (Supp. Table 3). Neither the Planctomycetota nor the Verrucomicrobiota encoded functions unique to either group as they were observed in other phylogenetic clades. The unclassified, putative Latescibacterota nGOM Bacteria 50, encoded 878 functions, one of which, relating to cell membrane biogenesis (LrgB-like family), was unique, not found in any other microbe in this study (Supp. Table 4). As noted, this microorganism was not definitively part of Latescibacterota, therefore core functions across this clade were not determined. The Proteobacteria clade core was comprised of 708 functions, 90 of which were statistically enriched in this group and 65 functions were found only in this clade, including rubredoxin, cobalamin synthesis, nitrate reductase delta subunit and several uncharacterized conserved proteins (Supp. Table 5).

### Methane oxidation pathway

#### Methane to methanol

The conversion of methane to methanol is mediated by methane monooxygenase (Fig. 1); therefore, *pmoA* (or sMMO) is often used as a marker gene for methanotrophs. Pfam and blastx annotations revealed that 34 genomes encoded *pmoA*, while the remaining four *Methylococcales* gene annotations were less clear, with blastx suggesting PmoA/AmoA were encoded while Pfam annotations revealed PmoC/AmoC were encoded in these genomes. Despite the discrepancy in the *Methylococcales* gene annotations based on the database searched, for the sake of clarity we will refer to the genes in this group as *pmoA*. Thus, all 38 microbes in this study encoded *pmoA* (Fig. 3 and Supp. Table 1), but none had the complete operon (*pmoA, pmoB* and *pmoC*), with the genes surrounding *pmoA* being annotated as hypothetical proteins. None of the 38 genomes coded for any part of the sMMO operon.

To determine *pmoA* abundance and expression, DNA and RNA reads were mapped to the 38 genomes. The Planctomycetota had the highest DNA RPKM for *pmoA* genes (2.2–8.1) of the *pmoA* in our study (Fig. 3). Verrucomicrobiota clade members *pmoA* also recruited DNA reads, but had lower DNA RPKM values (a maximum of 3.9) than the Planctomycetota (Fig. 3). The remaining Latescibacterota *pmoA* recruited few DNA reads (RPKM < 0.6), while Proteobacteria *pmoA* did not recruit any DNA reads (Fig. 3). Eleven genomes encoded the most highly expressed *pmoA* genes (the RNA RPKM range was 0.042–0.58) of all 38 microbes. These 11 genomes represented all clades, including the Proteobacteria, and three nGOM MAGs (nGOM Plancto 56, nGOM Bacteria 50 and nGOM Verruco 11) (Fig. 3).

#### Methanol to formaldehyde

No genes encoding MDH, which mediates the conversion of methanol to formaldehyde (Fig. 1), were identified in any of the genomes, including the canonical proteobacterial methanotrophs. However, every microbe in this study encoded NAD(P)-dependent dehydrogenase, in the SDR family (Fig. 3). SDR genes are present in a variety of organisms and are capable of oxidizing primary and secondary alcohols, including methanol, albeit with low affinity (Brändén et al. 1975, Arfman et al. 1997, Guo et al. 2019).

Of the 38 genomes, the nGOM Bacteria 50 SDR gene had the highest RPKM values compared with all other methane oxidation genes in this study. For this MAG, RPKM ranged from an average of 19.1 in hypoxic sites to an average of 2.98 in oxic sites, with a maximum DNA RPKM of 43.9 from site H_E4 (Fig. 3). Unlike DNA read recruitment, the nGOM Bacteria 50 SDR gene only recruited RNA reads from hypoxic samples (the RPKM range was 1.08–2.83) (Fig. 3). Some Planctomycetota SDR genes had similar DNA RPKM values to that of nGOM Bacteria 50. For example, the nGOM Plancto 56 SDR genes recruited DNA reads from oxic (RPKM max was 31.6) and hypoxic (RPKM max was 19.7) samples (Fig. 3). Planctomycetota SDR gene RNA read recruitment RPKM values were the highest of all 38 microbes, with oxic sample RPKM of up to 5.12 and hypoxic sample RPKM of up to 5.3 (Fig. 3). In the Verrucomicrobiota, the non-nGOM *Pedosphaera sp ARS72*SDR recruited the largest number of reads in this clade with average DNA RPKM values of 6.47 in oxic samples and 3.58 in hypoxic samples and the largest number of RNA reads, with an RPKM average of 1.11 in oxic sites and 0.23 RNA RPKM in hypoxic sites (Fig. 3). The remaining genomes recruited a lower number of DNA and RNA reads to SDR genes compared with those microbes discussed above (Fig. 3).

#### Formaldehyde to formate

Methanotrophs can employ multiple pathways for formaldehyde detoxification, assimilation and oxidation (Vorholt 2002, Chistoserdova et al. 2009) (Fig. 1). The most straightforward conversion of formaldehyde to formate is facilitated by formaldehyde dehydrogenase (FALDH), however, no FALDH genes were identified in COGs or Pfams annotations in any of the 38 microbes analyzed. Instead, a non-specific ALDH, which can be used to oxidize formaldehyde to formate (Patel et al. 1979, Anthony 1982) (Fig. 1), was identified as a core gene in all 38 genomes (discussed in detail below) (Fig. 3). Additional pathways to convert formaldehyde to formate include the tetrahydromethanopterin (H4MPT), the tetrahydrofolate (H4F) and the glutathione-dependent (GSH) pathway (Fig. 1), and are presented below. The H4MPT pathway has four steps: formaldehyde activating enzyme (FAE), tetrahydromethanopterin dehydrogenase (MtdB), tetrahydromethanopterin cyclohydrolase (Mch) and formylmethanofuran-tetrahydromethanopterin formyltransferase (Ftr/Fhc) (Fig. 1). The tetrahydrofolate (H4F) pathway is comprised of two main enzymes: the bifunctional methenyltetrahydrofolate cyclohydrolase/methylenetetrahydrofolate dehydrogenase (folD) and formate—tetrahydrofolate ligase (also called formyltetrahydrofolate synthetase; Fhs) (Fig. 1). The GSH includes glutathione-dependent formaldehyde activating enzyme (Gfa), glutathione-dependent formaldehyde dehydrogenase (GD-FALDH) and formyl-glutathione hydrolase (FGH) (Fig. 1).

#### ALDH pathway

The ALDH gene was expressed primarily in hypoxic sites, by all clades, except for Proteobacteria and Latescibacterota, whose ALDH genes did not recruit any DNA or RNA reads (Fig. 3). Planctomycetota ALDH genes recruited the most DNA reads compared with other clades in this study (Fig. 3). Specifically, Planctomycetota recruited the greatest number of DNA and RNA reads to a maximum of 8.19 DNA RPKM and 2.35 RNA RPKM (Fig. 3). The three nGOM Planctomycetota and Verrucomicrobiota ALDH genes recruited DNA and RNA reads, however, RPKM values were typically lower than those of the non-nGOM Planctomycetota (Fig. 3). The ALDH genes encoded in the remaining genomes recruited the fewest DNA and RNA reads. Proteobacterial ALDH genes not recruiting any DNA or RNA reads (Fig. 3).

##### Tetrahydromethanopterin (H4MPT) pathway

Planctomycetota and Proteobacteria clades encoded the full or partial H4MPT pathway (Fig. 3), but members of the other clades evaluated did not encode any part of the H4MPT pathway in their genomes (Fig. 3). *Rhodopirellula sp NAT69* and *Rhodopirellula sp SAT12* genes that are part of the H4MPT pathway recruited the greatest number of DNA and RNA reads (the RPKM maxima were 5.3 and 3.51, respectively), relative to the other microbes that encoded this full or partial pathway (Fig. 3). The nGOM Plancto 56, the only nGOM microbe that encoded a complete H4MPT pathway, had minimal DNA and RNA read recruitment to the genes in this pathway (RPKM < 1; (Fig. 3)), which is of the same order of magnitude as other Planctomycetota in this group (Fig. 3). While this full pathway was encoded in the Proteobacteria genomes analyzed herein, genes in this pathway did not recruit any DNA or RNA reads (Fig. 3).

##### Tetrahydrofolate (H4F) pathway

Of the 38 genomes, analyzed only three non-nGOM Latescibacterota encoded a complete H4F pathway (Fig. 3), with some members of the remaining clades encoding partial pathways. For the complete pathway, only the *Gemmatimonadetes bacterium RS822* genes in the H4F pathway recruited DNA reads, and this was from all sites except one (average oxic RPKM value of 0.04 and average hypoxic RPKM value of 0.135), but recruited RNA reads only from a hypoxic sample (RPKM was 0.03; Fig. 3). The remaining microbes encoded either a partial H4F pathway, or no genes in this pathway (Fig. 3). For those that encoded a partial pathway, six non-nGOM Planctomycetota H4F pathway genes recruited the greatest number of DNA reads compared with the other microbes that encoded this pathway (RPKM maximum value was 1.6), but no RNA reads were recruited (Fig. 3). The nGOM Bacteria 50 partial H4F pathway genes recruited a low number of DNA reads but had the highest RNA RPKM values for this pathway compared with other microbes, with average DNA RPKM of 0.63 and average RNA RPKM of 0.12 from hypoxic sites compared with oxic sites (average DNA RPKM was 0.13, average RNA RPKM was 0) (Fig. 3). Members of the other clades H4F pathway genes recruited a low number, or no DNA or RNA reads (Fig. 3).

##### Glutathione-dependent (GSH) pathway

Non-nGOM *Verrucomicrobiales bacterium SP5* and *Gemmatimonadetes bacterium NP81* genomes represented the only microbes analyzed herein that encoded two parts of the GSH pathway; however, no DNA or RNA reads were recruited (Fig. 3). Representatives of the Planctomycetota, Verrucomicrobiota, Latescibacterota and the Proteobacteria encoded only one part of the GSH pathway (Figs. 1 and 3). The Planctomycetota *Rhodopirellula sp NAT69* partial GSH pathway had the highest RPKM value for DNA read recruitment (RPKM maximum was 2.53) compared with the other microbes that encode this partial or full pathway (Fig. 3). The genes in this partial pathway of the remaining microbes recruited a low number or no DNA reads (Fig. 3). The only genes in the GSH pathway that were represented in the metatranscriptomic data were *Rhodopirellula sp SAT12*with RNA RPKM ranging from 0.053 to 0.54 (Fig. 3). None of 38 microbial genomes analyzed here encoded GD-FALDH.

### Formate to carbon dioxide

FDH catalyzes the conversion of formate directly to carbon dioxide (Anthony 1991, Dijkhuizen et al. 1992, Hanson and Hanson 1996) (Fig. 1). All Proterobacteria and most Latescibacterota encoded FDH, as did the two nGOM Verrucomicrobiota MAGs, and the unclassified nGOM Bacteria 50, but none of the Planctomycetota encoded FDH (Fig. 3). The nGOM Bacteria 50 FDH gene recruited DNA reads from each site (average oxic RPKM was 0.16 and average hypoxic RPKM was 0.98) and was the only one out of all 38 microbes whose FDH genes recruited any RNA reads (site H_E4 only with an RPKM of 0.21; Fig. 3). The nGOM Verruco 11 and *Pedosphaera sp ARS72* FDH genes recruited DNA reads from all sites (RPKM range was 0.06–0.39) (Fig. 3). The remaining Verrucomicrobiota that encoded FDH recruited a low number of DNA reads, while no DNA or RNA reads were recruited to this gene encoded in Latescibacterota or Proteobacteria (Fig. 3).

### CO_2_ fixation

A key gene in autotrophic carbon dioxide fixation is ribulose-1,5-bisphosphate carboxylase/oxygenase (RuBisCO) and some more recently discovered methanotrophs classified as Verrucomicrobiota and NC10 have been shown to encode this gene (Khadem et al. 2011, Rasigraf et al. 2014). For example, the analysis by Thrash et al. (2017) identified this functional annotation in nGOM Bacteria 50 using IMG. According to annotations by both COGs and Pfams in this study, this gene was encoded by nGOM Bacteria 50 and some of the Latescibacterota (*Gemmatimonadetes bacterium RS821, Gemmatimonadetes bacterium RS822, Gemmatimonadetes bacterium SAT128* and *Latescibacteria bacterium UBA6620*), but these genes recruited few (RPKM < 0.8) if any DNA reads and no RNA reads in any sample.

## Discussion

The average methane concentrations and oxidation rates in the 2013 dead zone (Rogener et al. 2021) were well above background levels of <4 nM (Joye et al. 2011) and 0.05 nmol L^−1^d^−1^ (Crespo-Medina et al. 2014) observed in the offshore GOM. Specifically, Rogener et al. (2021) reported depth-integrated methane oxidation rates (Fmox) of 0.2 µmols m^−2^d^−1^ in 2013. They also reported that methane concentrations in the nGOM were negatively correlated with O_2_ concentrations, which have been previously described (Abril and Iversen 2002, Kelley 2003, Mau et al. 2013, Osudar et al. 2015, Steinle et al. 2017, Rogener et al. 2021). Gillies et al. (2015) and Campbell et al. (2019) datasets revealed that relative proportions of canonical methanotrophs were very low. Rogener et al. (2021) also made measurements in the 2015 dead zone and reported Fmox of 2.4 µmols m^−2^d^−1^. In parallel, 2015 dead zone samples, Campbell and Mason (unpublished) used iTag sequencing to evaluate the microbial community in the 2015 dead zone and found Methylococcales, which contain canonical marine methanotrophs, reached a maximum abundance of 1% of the community, averaging 0.09% relative abundance. *Methylosinus* was even less abundant, averaging 0.003% in relative abundance. Given that the abundance of canonical methanotrophs was very low and invariant during 2013–2015, but Fmox was significantly higher in 2015, the data suggest that unknown, non-canonical methanotrophs are likely carrying out methane oxidation in this shallow water, methane-rich environment.

The microbes we presented herein with genomes encoding partial to complete pathways for methane oxidation are united in that they represent non-canonical aerobic, largely marine methanotrophs, which are not known to mediate methane oxidation. The presence of these novel methanotrophs in the nGOM dead zone begins to reconcile the high methane oxidation rates that have been reported in this ecosystem (Rogener et al. 2021) with the low to undetectable levels of canonical methanotrophs (Gillies et al. 2015, Campbell et al. 2019 and Campbell and Mason unpublished data). The level of DNA and RNA read recruitment to these genomes and genes was highest for nGOM MAGs, which is not unexpected. The lower read recruitment to non-nGOM microbes suggested that while these microbes were not abundant or highly active in the nGOM dead zone at the time we sampled, they encode a previously unrecognized capacity to oxidize methane in a diversity of marine environments, in which they may be both abundant and actively consuming methane.

These 38 methanotrophs, both from the nGOM and outside of the GOM, were further united in that beyond *pmoA* they all have the metabolic capacity for methanol and formaldehyde oxidation (SDR and ALDH). Additional modes of formaldehyde oxidation (H4MPT, H4F, GSH) were also detected, but these pathways were incomplete in most genomes, if they were encoded at all. Further, all clades except Planctomycetota, contained at least some members encoding FDH. As expected, the canonical methanotrophs in the Proteobacteria encoded complete methane oxidation pathways. In addition, the non-canonical methanotrophs, including the unclassified nGOM MAG, as well as most Latescibacterota and Verrucomicrobiota in this study, encoded complete methane oxidation pathways, while all Planctomycetota encoded partial methane oxidation pathways. The partial to complete methane oxidation pathways encoded in these non-canonical planktonic methanotrophs reflect the phylogenetic diversity of methanotrophs, as well as the potential role in mitigating methane efflux from the ocean.

All the Planctomycetota genomes encoded partial MOx pathways, with their *pmoA* genes recruiting more RNA reads in oxic sites than hypoxic. The Planctomycetota nGOM and global microbes analyzed herein encoded the full or partial H4MPT pathway and partial H4F pathway (Figs. 1 and 3), which is consistent with previous reports for members of this phylogenetic clade (Chistoserdova et al. 2004, Woebken et al. 2007, Fuerst and Sagulenko 2011). The nGOM Planctomycetota MAGs and the majority of global Planctomycetota genomes encoded a partial GSH pathway (FGH) (Figs. 1 and 3), as has been previously reported (Woebken et al. 2007). Not all Planctomycetota encode FDH (Kim et al. 2016), and none of the nGOM or global Planctomycetota in this study did. While the three Planctomycetota from non-marine environments (*Planctomyces sp SHPL14, Planctomycetaceae bacterium Baikal G1 2R* and *Rubinisphaera brasiliensis DSM5305*) encoded enough modules to complete the methane oxidation pathway to formate (because none encode FDH), they do not appear to be actively carrying out methane oxidation in this environment (Fig. 3).

The nGOM MAGs and two microbes from the Arabian Sea (Tully et al. 2018) recruited the most DNA and RNA reads to their genomes compared with other Planctomycetota (Fig. 3). Both ecosystems experience hydrocarbon contamination and low oxygen concentrations (temporary in the case of nGOM, permanent in the Arabian Sea), and it appears these microbes encode similar modules of the methane oxidation pathway (Fig. 3). Members of this clade contain genes involved in C1 transfer pathways (H4MPT, H4F) and although the potential for methylotrophy has been suggested (Buckley et al. 2006), thus far they have only been hypothesized to be key ancestral players in the evolution of the global methane cycle (Glöckner et al. 2003, Bauer et al. 2004, Chistoserdova et al. 2004, Kalyuzhnaya et al. 2004, Chistoserdova et al. 2005, Woebken et al. 2007). This diverse clade is found in numerous environments and is one of the most abundant groups in OMZs (Wright et al. 2012), where they are primary mediators of nitrogen loss through anaerobic oxidation of ammonium (Kuypers et al. 2003, 2005, Schmid et al. 2007, Woebken et al. 2008, Galán et al. 2009, Lam et al. 2016). Our study results suggest a new function for Planctomycetota in OMZs: methane oxidation, which expands their role in biogeochemical cycles and supports the previously hypothesized role of this clade in global methane cycles.

Verrucomicrobiota are also common in OMZs (Wright et al. 2012), but are not known to carry out methane oxidation in the marine environment. The Verrucomicrobiota we analyzed were actively expressing *pmoA* in low oxygen environments, which suggested they may be like the thermoacidophilic verrucomicrobial methanotrophs that oxidize methane, but in acidic, geothermal environments (Dunfield et al. 2007; Pol et al. 2007; Islam et al. 2008). These methanotrophic Verrucomicrobiota do not employ the same MOx pathway modules that are observed in canonical (Proteobacteria) methanotrophs. For example, they have been shown to code for XoxF-MDH, which converts methanol directly to formate (Keltjens et al. 2014), thereby lacking common formaldehyde oxidation modules (Dunfield et al. 2007). XoxF-MDH was not annotated in the Verrucomicrobiota genomes analyzed here (only SDR), nor was a more common, complete formaldehyde oxidation pathway (H4MPT, H4F, GSH).

Acidophilic verrucomicrobial methanotrophs have been shown to encode the complete H4F pathway (Picone et al. 2021, Schmitz et al. 2021), but none in this study did, only a partial one if at all. Thus, the formaldehyde oxidation step remains unclear in the Verrucomicrobiota analyzed herein. The last step in methane oxidation encoded by FDH was annotated in five of the seven Verrucomicrobiota, which is consistent with acidophilic terrestrial methanotrophs (Dunfield et al. 2007, Picone et al. 2021, Schmitz et al. 2021). *Pedosphaera sp ARS72*, which is from a site in the Arabian Sea where annual mean oxygen is <2.66 mg L^−1^ (Pesant et al. 2015), and nGOM Verruco 11, both encode the same modules of the methane oxidation pathway (except H4F) (Fig. 3). These microorganisms are from areas that experience hydrocarbon contamination and low oxygen concentrations and were some of the most abundant (Fig. 2) and active microbes in this study (Fig. 3). Thus, the Verrucomicrobiota presented herein are like their terrestrial, acidophilic relatives in that they can carry out methane oxidation, but do so in a basic, aquatic marine environment where they may act as an important methane biofilter in the water column using novel modules within the overall methane oxidation pathway (Dunfield et al. 2007).

Of the nine Latescibacterota in this study, eight were originally classified as Gemmatimonadetes. Members of the Gemmatimonadetes (now classified as either Gemmatimonadota or Latescibacterota) are also abundant in OMZs, although typically less so than Planctomycetota (Wright et al. 2012), and while not recognized as aerobic marine methanotrophs, some genomes have been shown to encode MDH, classifying them as methylotrophs (Butterfield et al. 2016). Members of Latescibacterota (previously WS3 and candidate phylum Lastescibacteria) were first discovered in a hydrocarbon-contaminated aquifer and have since been found in marine sediments, hydrothermal vents, soil and other hydrocarbon-contaminated environments (Farag et al. 2017). Recently, members of this clade were shown to encode for fermentation (García-Lozano et al. 2019) and anaerobic hydrocarbon degradation (Dombrowski et al. 2017), still none have been reported to encode the capacity for aerobic methane oxidation. The Latescibacterota in this study all have *pmoA*, SDR and ALDH, although most of those genes recruited minimal DNA and RNA reads. These microbes did not encode MDH or any part of the H4MPT pathway, which contrasts with previous research (Butterfield et al. 2016); however, Butterfield et al. (2016) did not find that their microbes encoded *pmoA*. Most of the Latescibacterota in this study encoded a partial H4F pathway, but few encoded the GSH pathway. Despite the presence of *pmoA* and other modules in MOx, Latescibacterota did not appear to be active in MOx in the nGOM. However, the Latescibacterota presented here represent the first aerobic methane oxidizing members of this clade in the marine environment. It is possible that at other timepoints in the nGOM, or in other environments with different methane or oxygen concentrations, this group of bacteria could become active and serve as a methane biofilter.

Unlike the previously discussed taxa, the Proteobacteria contain canonical aerobic marine methanotrophs (Hanson and Hanson 1996). This clade has been reported as the most abundant group in global OMZs via 16S rRNA gene surveys (Wright et al. 2012). All four Proteobacteria in this study encoded *pmoA*, SDR and ALDH, but genomic and transcriptomic read representation was minimal. They also all encoded the full H4MPT pathway, partial H4F and GSH pathways and all encoded FDH. While these microbes represented canonical methanotrophs, like the other 34 genomes analyzed herein, they lacked MDH genes, thus their genomes encoded only some of typical modules to carry out MOx. These canonical methanotrophs were not abundant and or had low levels of activity in the nGOM hypoxic zone, where methane concentrations and oxidation rates are high, thus highlighting the importance of novel, non-canonical methanotrophs potentially acting as a biofilter.

## Conclusion

Climate change-induced expansion of OMZs that are enriched in greenhouse gases suggests an active planktonic methanotrophic community is even more critical in mitigating greenhouse gas efflux from the water column to the atmosphere. Herein we show that canonical methanotrophs (Proteobacteria) appear to be less abundant and active in the 2013 nGOM dead zone than previously unrecognized methanotrophs belonging to Verrucomicrobiota, Planctomycetota and Latescibacterota. These non-canonical methanotrophs are globally distributed and may play a key role in oxidizing methane in the water column before it reaches the atmosphere. Further research should investigate the alternative modes of formaldehyde and formate oxidation by these uncultivated, non-canonical aerobic marine methanotrophs. Further, methanol oxidation via MDH was not encoded in any of 38 genomes, including the canonical methanotrophs. Thus, additional analyses are needed to better understand the metabolic capacities and potential to act as a methane biofilter by canonical and non-canonical methanotrophs, across an oxygen gradient, as well as to re-evaluate the marine microbial methane sink. Further, this study provides an illustration on why assessing taxonomy alone (e.g. 16S rRNA gene data) may obscure important metabolic processes that are revealed using multi-omics approaches. Finally, in the age of omics, our analyses are likely the first of many in which new, non-canonical methanotrophs are revealed.

## Supplementary Material

fiac153_Supplemental_Files
