# Supplementary material for: Metagenomics and metatranscriptomics reveal broadly distributed, active, novel methanotrophs in the Gulf of Mexico hypoxic zone and in the marine water column
Source: FEMS Microbiol Ecol. 2022 Dec 15;99(2):fiac153. doi: 10.1093/femsec/fiac153 (PMC9874027; doi:10.1093/femsec/fiac153)
Supplement: fiac153_Supplemental_Files [file fiac153_supplemental_files.zip › Supp_data_Figure_1_map.pdf]

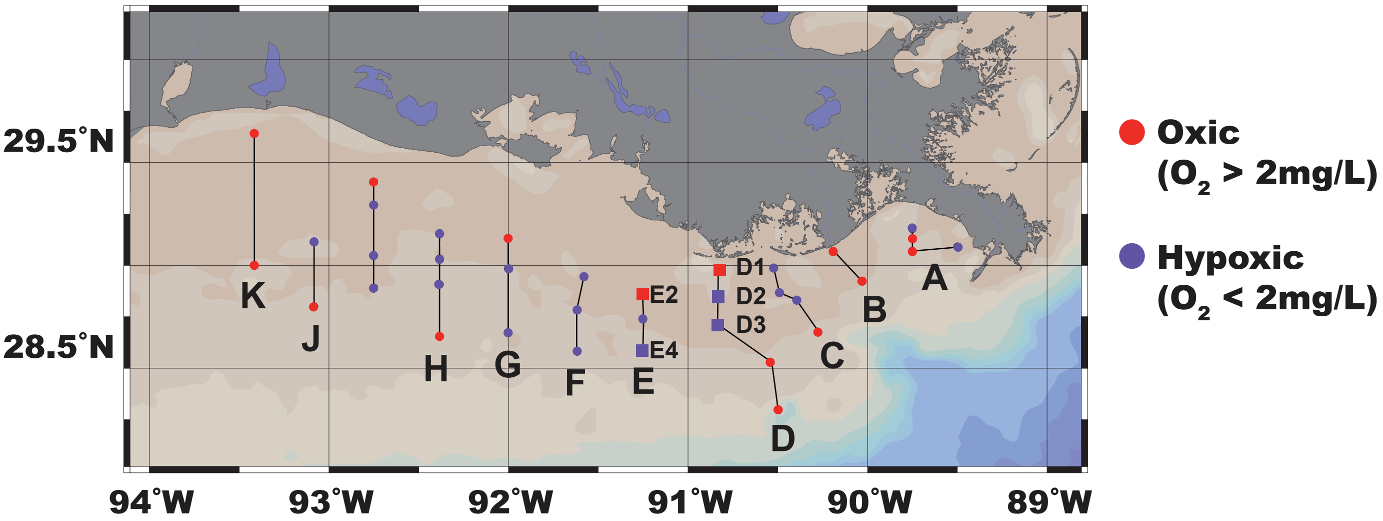

Supplemental Figure 1. Map from Gillies et al. (2015) of all samples collected in the 2013 nGOM dead zone. Samples selected for metagenomic and metatranscriptomic sequencing, O_D1, H_D2, H_D3, O_E2, and H_E4, are labelled.
