# Supplementary material for: Metagenomics and metatranscriptomics reveal broadly distributed, active, novel methanotrophs in the Gulf of Mexico hypoxic zone and in the marine water column
Source: FEMS Microbiol Ecol. 2022 Dec 15;99(2):fiac153. doi: 10.1093/femsec/fiac153 (PMC9874027; doi:10.1093/femsec/fiac153)
Supplement: fiac153_Supplemental_Files [file fiac153_supplemental_files.zip › Supp_data_Figure_2_tree_111722.pdf]

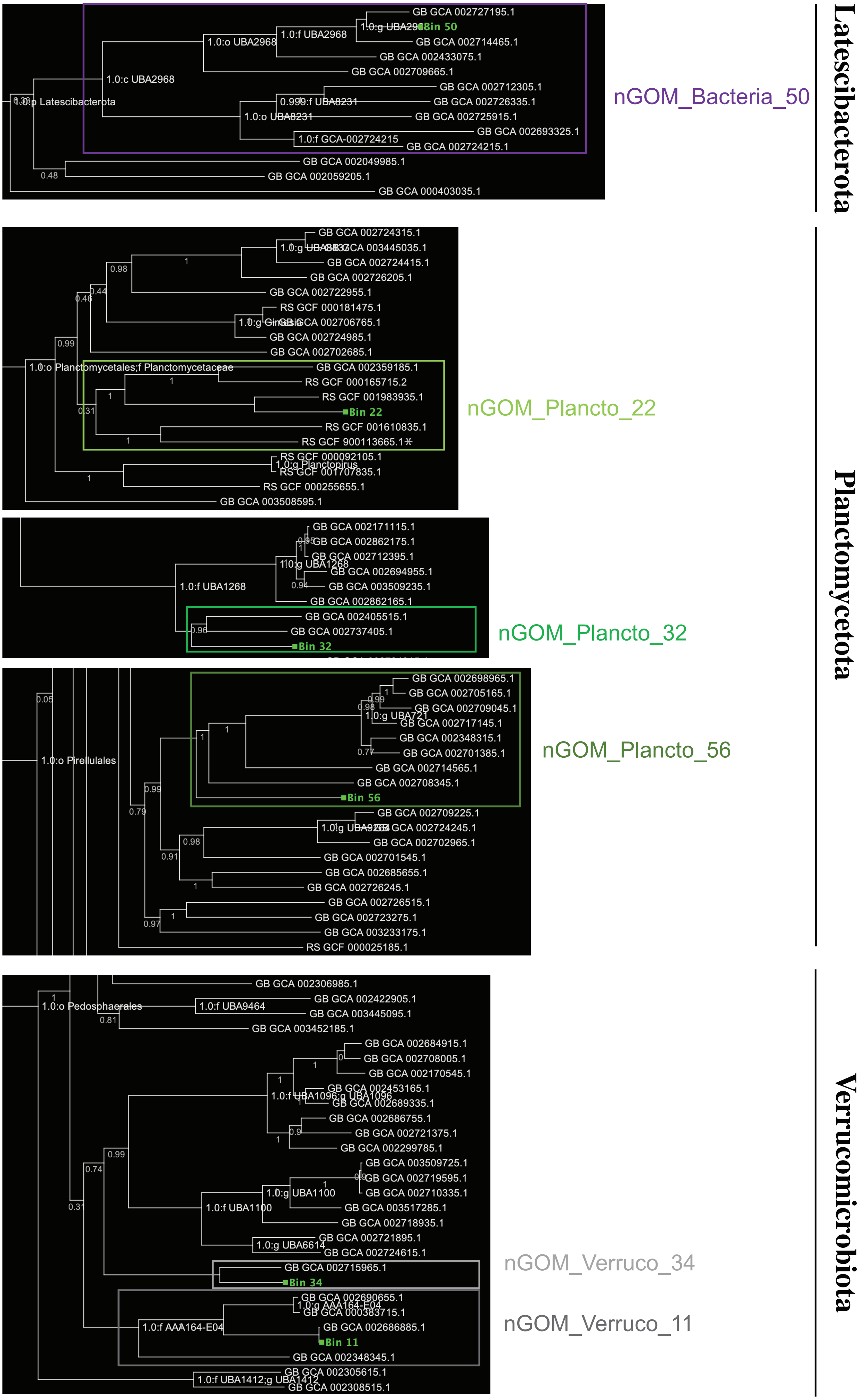


**Supplemental Figure 2.**

Ribosomal protein phylogenetic tree from GTDB showing only relevant microbes, not all 46,926 nodes in the original tree, with boxes denoting close relatives of the nGOM MAGs. The asterisk indicates a microbe that was a close relative to nGOM MAGs, but its genomic data was restricted, so it was not included in any analyses. The four canonical methanotrophs included in our analyses were not closely related to the nGOM MAGs, so are not shown in this figure.

**Supplemental Figure 2.** Ribosomal protein phylogenetic tree with boxes denoting close relatives of the nGOM MAGs used in downstream analysis. The asterisk indicates a microbe that was a close relative to nGOM MAGs, but its genomic data was restricted, so it was not included in any analyses. The four canonical methanotrophs included in our analyses were not closely related to the nGOM MAGs, so are not shown in this figure.
